# Ferumoxytol enhanced black-blood cardiovascular magnetic resonance imaging

**DOI:** 10.1186/s12968-017-0422-y

**Published:** 2017-12-28

**Authors:** Kim-Lien Nguyen, Eun-Ah Park, Takegawa Yoshida, Peng Hu, J. Paul Finn

**Affiliations:** 1 Diagnostic Cardiovascular Imaging Laboratory, Department of Radiological Sciences, David Geffen School of Medicine at UCLA, Los Angeles, California USA; 2 Division of Cardiology, David Geffen School of Medicine at UCLA and VA Greater Los Angeles Healthcare System, Los Angeles, California USA; 3 Physics and Biology in Medicine Interdepartmental Graduate Program, Department of Radiological Sciences, University of California at Los Angeles, Peter V. Ueberroth Building Suite 3371, 10945 Le Conte Ave, Los Angeles, CA 90095-7206 USA; 4 Department of Radiology and The Institute of Radiation Medicine, Seoul National University Hospital, Seoul, 110-744 South Korea

**Keywords:** USPIO, Ferumoxytol, Magnetic resonance imaging, Black-blood imaging, HASTE

## Abstract

**Background:**

Bright-blood and black-blood cardiovascular magnetic resonance (CMR) techniques are frequently employed together during a clinical exam because of their complementary features. While valuable, existing black-blood CMR approaches are flow dependent and prone to failure. We aim to assess the effectiveness and reliability of ferumoxytol enhanced (FE) Half-Fourier Single-shot Turbo Spin-echo (HASTE) imaging without magnetization preparation pulses to yield uniform intra-luminal blood signal suppression by comparing FE-HASTE with pre-ferumoxytol HASTE imaging.

**Methods:**

This study was IRB-approved and HIPAA compliant. Consecutive patients who were referred for FE-CMR between June 2013 and February 2017 were enrolled. Qualitative image scores reflecting the degree and reliability of blood signal suppression were based on a 3-point Likert scale, with 3 reflecting perfect suppression. For quantitative evaluation, homogeneity indices (defined as standard deviation of the left atrial signal intensity) and signal-to-noise ratios (SNR) for vascular lumens and cardiac chambers were measured.

**Results:**

Of the 340 unique patients who underwent FE-CMR, HASTE was performed in 257. Ninety-three patients had both pre-ferumoxytol HASTE and FE-HASTE, and were included in this analysis. Qualitative image scores reflecting the degree and reliability of blood signal suppression were significantly higher for FE-HASTE images (2.9 [IQR 2.8–3.0] vs 1.8 [IQR 1.6–2.1], *p* < 0.001). Inter-reader agreement was moderate (k = 0.50, 95% CI 0.45–0.55). Blood signal suppression was more complete on FE-HASTE images than on pre-ferumoxytol HASTE, as indicated by lower mean homogeneity indices (24.5 [IQR 18.0–32.8] vs 108.0 [IQR 65.0–170.4], *p* < 0.001) and lower blood pool SNR for all regions (5.6 [IQR 3.2–10.0] vs 21.5 [IQR 12.5–39.4], *p* < 0.001).

**Conclusion:**

FE-HASTE black-blood imaging offers an effective, reliable, and simple approach for flow independent blood signal suppression. The technique holds promise as a fast and routine complement to bright-blood cardiovascular imaging with ferumoxytol.

**Electronic supplementary material:**

The online version of this article (10.1186/s12968-017-0422-y) contains supplementary material, which is available to authorized users.

## Background

Black-blood cardiovascular magnetic resonance (CMR) imaging is important for depiction of vessel wall and intra-cardiac abnormalities. While the intensity and uniformity of luminal enhancement serve as the benchmark for bright blood CMR angiography, the completeness and uniformity of blood pool signal suppression set the complementary standard for black-blood imaging. A variety of strategies have been employed to suppress the blood signal [1], but most are to some degree flow dependent [2]. As a result, slow or inconsistent blood flow can result in persistence of signal within a patent vessel or cardiac chamber, mimicking a mass or thrombus.

Because of its speed, simplicity, and resistance to motion artifact, Half-Fourier Single-shot Turbo Spin-echo (HASTE) [3] imaging is widely used to complement bright blood techniques in CMR imaging [1]. The long echo train of HASTE (and related multi-echo spin echo techniques) supports efficient signal suppression for moderate to high velocity flow, but signal from slowly flowing blood can persist. For this reason, a dual inversion recovery magnetization preparation scheme is typically used to null the magnetization of blood which enters the imaging slice between the inversion and the readout [4]. Dual inversion recovery is effective as an adjunct for many black-blood applications and remains the most widely used scheme for black-blood conditioning of the great vessels and cardiac chambers. However, because of variable inflow speeds, electrocardiographic (ECG) gating with patient-specific adjustment of timing and imaging parameters may be necessary. Furthermore, for certain slice orientations and imaging parameters, timing conflicts within the cardiac cycle may undermine achievable image resolution or blood suppression, particularly in vessels with slow flow. Therefore, a flow-independent black-blood CMR technique would be desirable.

Recently, ferumoxytol has re-surfaced as an attractive CMR contrast agent [5, 6]. Although ferumoxytol is approved by the U.S. Food and Drug Administration (FDA) for intravenous treatment of iron deficiency anemia in adults with kidney disease, its pharmacokinetics and potent r_1_ and r_2_ effects (*r*_1_ = 15 mM^−1^ s^−1^, *r*_2_ = 89 mM^−1^ s-^1^ at 1.5 T) [6, 7] can be exploited to support both bright blood angiography and flow independent, post-contrast black-blood imaging.

The purpose of our study is to evaluate the potential of ferumoxytol to generate reliable, flow independent, black-blood HASTE images, as a complement to bright-blood CMR angiography of the thorax, without the need for any magnetization preparation schemes, by comparing it with pre-ferumoxytol HASTE imaging.

## Methods

### Study population

**Fig. 1:**
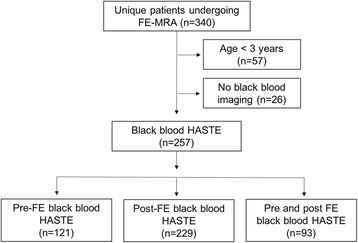
Flowchart of study population

This is a retrospective analysis of prospectively enrolled patients. All study procedures were approved by the local institutional review board and were compliant with the Health Insurance Portability and Accountability Act. Written informed consent was obtained from all patients or from their legal guardians. Consecutive patients age ≥ 3 years who were referred for ferumoxytol-enhanced (FE) magnetic resonance angiogram  (MRA) from June 2013 to February 2017 and who had black-blood CMR were enrolled (*n* = 257). Figure 1 outlines the final patient population included in the analysis.

### Image acquisition

Examinations were performed either on a 3 T (TIM Trio [*n* = 161], Prisma Fit [*n* = 42], Skyra [*n* = 23]; Siemens Healthineers, Erlangen, Germany) or a 1.5 T (Avanto [*n* = 31]; Siemens Healthineers) whole-body system. A combination of phased array body coils, spine coils, head and neck coils or extremity coils were used depending on patient size.

Following initial localizer images, non-breath held HASTE images were acquired in the coronal plane [1, 8]. Typical acquisition parameters are outlined in Table 1. A dual inversion recovery, black blood magnetization preparation scheme was employed, but in most cases without ECG gating, such that the inflow time was not synchronized with or consistent within the cardiac cycle. The time between adjacent slice acquisitions (repetition time) was fixed at 3.0 s at 1.5 T and 4.0 s at 3 T. The reason for the longer than minimum repetition time was to allow for magnetization recovery within all tissues and to prevent cross talk and potential saturation between moving structures of adjacent slices. In one patient with a suspected left atrial mass on echocardiography, ECG gated pre-ferumoxytol HASTE imaging was performed in multiple planes, followed by post-ferumoxytol HASTE imaging. Typical slice thickness in adults was 7 mm at 1.5 T and 6 mm at 3 T. Stock ferumoxytol was diluted 6X –10X and administered as a slow infusion to a total dose of 4 mg /kg, as previously described [9]. Subsequently, breath-held, bright-blood, three-dimensional (3D) CMR angiography was acquired during the steady state distribution of ferumoxytol. FE-HASTE images were acquired with identical parameters to the pre-ferumoxytol HASTE images, but without the dual inversion magnetization preparation scheme. All black-blood images were acquired during quiet breathing.

**Table 1:** Representative technical parameters for coronal HASTE

	3 Tesla	1.5 Tesla
Echo spacing (ms)	4.22	4.04
Echo time (ms)	68	51
Echo train length	123	98
Flip angle (°)	160	160
Bandwidth (Hz)	574	751
Acquisition matrix	512 × 374	512 × 358
Field of view (mm^2^)	500 × 374	500 × 378
Slice thickness (mm)^a^	6	7
In-plane resolution (mm^2^)	1.0 × 1.1	1.0 × 1.4

^a^For small children, the similar parameters were used with an effective slice thickness of 4 mm without interslice gaps

### Image analysis

To qualitatively assess the effectiveness and reliability of blood signal suppression, two readers (T.Y. and E.A.P, each with 2 and 11 years of cardiovascular imaging experience, respectively) independently reviewed the pre-ferumoxytol and FE-HASTE images using a commercially available software platform (OsiriX, Pixmeo, Bernex, Switzerland). Images were de-identified and presented to reviewers in random order. The datasets were scored using a 3-point Likert grading scale: 3 = complete suppression of the blood signal within the vascular lumen on all slices; 2 = persistent, bright-blood signal within some part of the lumen on one or more slices; and 1 = persistent, bright-blood signal within a considerable part of the lumen on many or all slices. Ten cardiovascular regions within the chest cavity were scored: superior vena cava, both atria, both ventricles, the pulmonary trunk, the right and left pulmonary arteries, and the mid-ascending and mid-descending thoracic aorta. A final image quality score was calculated for each region by averaging the results of primary readers. In cases where the scores differed by 2 points between reader 1 and 2, a third reader (K.L.N., 5 years cardiovascular imaging experience) served as the consensus reader.

In patients with both pre-ferumoxytol and FE-HASTE images (*n* = 93), quantitative proxies of blood signal suppression were determined by calculating the signal-to-noise ratios (SNR) and generating a homogeneity index for each image dataset. Circular regions of interest (ROIs) measuring at least 1.0 cm^2^ were drawn in the blood pool of the vascular lumen on coronal images. Coronal HASTE image datasets were chosen because 1) all 93 patients had at least coronal HASTE datasets and 2) the coronal datasets represent the orientation in which all of the cardiovascular ROIs were included. Ten representative vascular and intra-cardiac regions were scored: superior vena cava, both atria, both ventricles, pulmonary trunk, right and left pulmonary arteries, ascending aorta, and descending thoracic aorta. Image noise was determined by averaging the standard deviation of four ROIs drawn in the background air located within the image field of view. SNRs for each region were calculated by dividing signal intensities by image noise. The homogeneity index reflecting blood signal suppression was defined as the standard deviation of signal in the left atrium. Lower homogeneity indices reflect greater homogeneous suppression of blood signal in the vascular lumen. The contrast-to-noise ratio (CNR) was calculated as the difference between the mean left ventricular signal intensity of the blood pool and myocardium divided by the standard deviation of noise in air.

### Statistical analysis

Data were tested for normality using the D’Agostino-Pearson test and log transformed where needed. Descriptive statistics are expressed as means and standard deviations (SD) or medians and interquartiles (IQR) or as absolutes and percentages. The SNR for both HASTE datasets were compared using analysis of variance (ANOVA) for repeated measurements. Image noise and CNRs were compared using the Wilcoxon-signed rank sum test for paired samples. To evaluate for inter-reader agreement, a kappa (k) value was calculated for qualitative image scores: poor (k < 0.20), fair (k = 0.21–0.40), moderate (k = 0.41–0.60), good (k = 0.61–0.80), and excellent (k = 0.81–1.00). Analyses were performed using MedCalc (Version 16.8.4, Belgium). Two-tailed *p* values less than 0.05 were considered statistically significant and Bonferroni corrected where appropriate.

## Results

Of the 340 patients who underwent FE-CMR between June 2013 to February 2017, a total of 257 patients (age 49 [IQR 16–67] years, 46% females) were ≥3 years old and had black-blood HASTE imaging. Ninety-three patients had both pre-ferumoxytol and FE-HASTE imaging. Characterization of the study population and clinical indications are outlined in Fig. 1 and Table 2, respectively. All studies were completed successfully and without major adverse events.

**Table 2:** Patient characteristics and clinical indications^a^

	All with HASTE (*n* = 257)	Pre-FE HASTE (*n* = 121)	Post-FE HASTE (*n* = 229)	Pre- and post-FE HASTE (*n* = 93)
Age, y	49 (16–67)	39 (13–65)	48 (16–67)	44 (16–67)
Gender (female)	118 (46%)	52 (43%)	95 (41%)	40 (43%)
Pre-CMR creatinine (mg/dL)	1.9 (1.2–2.9)	1.9 (1.0–3.0)	1.9 (0.99–2.8)	1.7 (0.95–2.85)
Clinical indications				
Aneurysm /dissection	18 (8%)	6 (5%)	18 (8%)	6 (6%)
AVMs	2 (1%)	0	2 (1%)	0
CHD	40 (16%)	15 (12%)	36 (16%)	11 (12%)
Embolus /thrombus	15 (6%)	7 (6%)	14 (6%)	6 (6%)
Interventional planning	28 (11%)	16 (13%)	26 (11%)	14 (15%)
Mass	19 (7%)	13 (11%)	19 (8%)	13 (14%)
Other	10 (4%)	4 (3%)	9 (4%)	3 (3%)
Placenta	4 (2%)	4 (3%)	4 (2%)	4 (4%)
Post-renal transplant	21 (8%)	12 (10%)	20 (9%)	11 (12%)
Pre-renal transplant	12 (5%)	6 (5%)	8 (3%)	2 (2%)
Vascular mapping	22 (9%)	12 (10%)	14 (6%)	4 (4%)
Vascular thrombosis	66 (26%)	26 (21%)	59 (26%)	19 (20%)

*AVMs* arteriovenous malformations, *CHD* congenital heart disease, *CKD* chronic kidney disease, *eGFR* estimated glomerular filtration rate, *FE* ferumoxytol enhanced, *y* years

^a^Values are reported as median and interquartile range or absolutes and frequencies

### Qualitative analysis

**Fig. 2:**
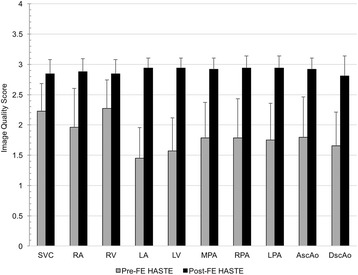
Qualitative comparison between pre-ferumoxytol HASTE and FE-HASTE images. A 3-point Likert grading scale was employed: 3 = complete suppression of the blood signal within the vascular lumen on all slices; 2 = persistent, bright blood signal within some part of the lumen on one or more slices; and 1 = persistent, bright blood signal within a considerable part of the lumen on many or all slices. Compared to pre-ferumoxytol HASTE images, the mean image quality scores for all 10 cardiovascular regions on FE-HASTE images were significantly higher (*p* < 0.001). *AscAo* ascending aorta; *DscAo* descending aorta; *LA* left atrium; *LPA* left pulmonary artery; *LV* left ventricle; *MPA* main pulmonary artery; *RA* right atrium; *RPA* right pulmonary artery; *RV* right ventricle; *SVC* superior vena cava

Compared to pre-ferumoxytol HASTE, the image quality scores reflecting effectiveness and reliability of blood signal suppression for FE-HASTE images were significantly higher (*p* < 0.001, Fig. 2). Using a 3-point Likert scale with grade 3 representing complete and uniform blood signal suppression, FE- HASTE images had an overall image quality score of 2.9 (IQR 2.8–3.0). The overall image quality score for pre-ferumoxytol HASTE was 1.8 (IQR 1.6–2.1). Inter-reader agreement was moderate (k = 0.50, 95% CI 0.45–0.55).

**Fig. 3:**
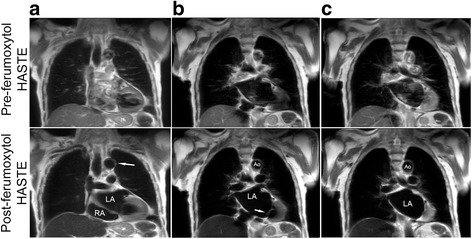
Pre- and post-ferumoxytol coronal CMR images (3 T) belonging to a 94-year old male with critical aortic stenosis who had vascular mapping prior to transcatheter aortic valve replacement. Pre-ferumoxytol HASTE images (**a**-**c**, upper panel) illustrate poor blood signal suppression. FE-HASTE images (**a**-**c**, lower panel) at approximately the same region demonstrate uniform blood signal suppression in all vascular regions and intra-cardiac chambers within the imaged field of view. A multi-slice comparison of pre-ferumoxytol HASTE and FE-HASTE images is available as Additional file 1: Video S1. Thin scallops of the mitral valve (**b**, lower panel) are well depicted against the uniformly dark left atrium. Atherosclerotic plaques along the vessel wall of the transverse aortic arch (**a**, lower panel, white arrow) are present. *Ao* aorta; *LA* left atrium; *RA* right atrium

**Fig. 4:**
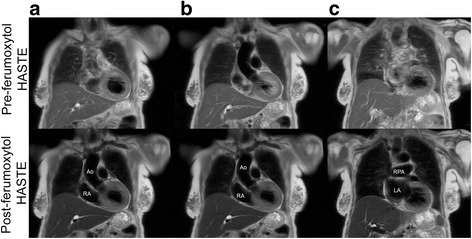
Pre and post-ferumoxytol coronal CMR images (3 T) belonging to a 93-year-old female with critical aortic stenosis who had FE-CMR for vascular mapping in the setting of renal impairment. Pre-ferumoxytol HASTE images (image quality score 1–2) illustrate inconsistent blood signal suppression across multiple slices (upper panel, **a-c**). The blood signal in FE HASTE images (image quality 3) is consistently and uniformly suppressed in both vascular lumen and intra-cardiac chambers and across multiple slices (lower panel, **a-c**) compared to pre-ferumoxytol HASTE images. *Ao* aorta; *LA* left atrium; *RA* right atrium; *RPA* right pulmonary artery

Figures 3 and 4 provide comparative multislice examples of pre-ferumoxytol HASTE and FE-HASTE images. Both patients in Figs. 3 and 4 have critical aortic stenosis and had FE-CMR in the setting of renal impairment. Pre-ferumoxytol HASTE images (Fig. 3 and Fig. 4, upper panel, a-c) show incomplete and variable luminal signal suppression across all three coronal slices. In contrast, FE-HASTE images (Fig. 3 and Fig. 4, lower panel, a-c) demonstrate uniform and complete luminal blood signal suppression across multiple slices. Complex atherosclerotic plaques can be confidently visualized on T2-weighted FE-HASTE image (Fig. 3a, lower panel, white arrow). Despite the severely dilated left atrium and differences in the flow pattern between the great vessels and the left atrium, there is uniform blood signal suppression on FE-HASTE images in all structures (Fig. 3, lower panel, a-c). Note the sharp left atrial myocardial edge definition on FE-HASTE images (Fig. 3b-c, lower panel). Although there is slight blurring of the myocardial edge in Fig. 4 (lower panel), the luminal blood signal suppression is uniform.

**Fig. 5:**
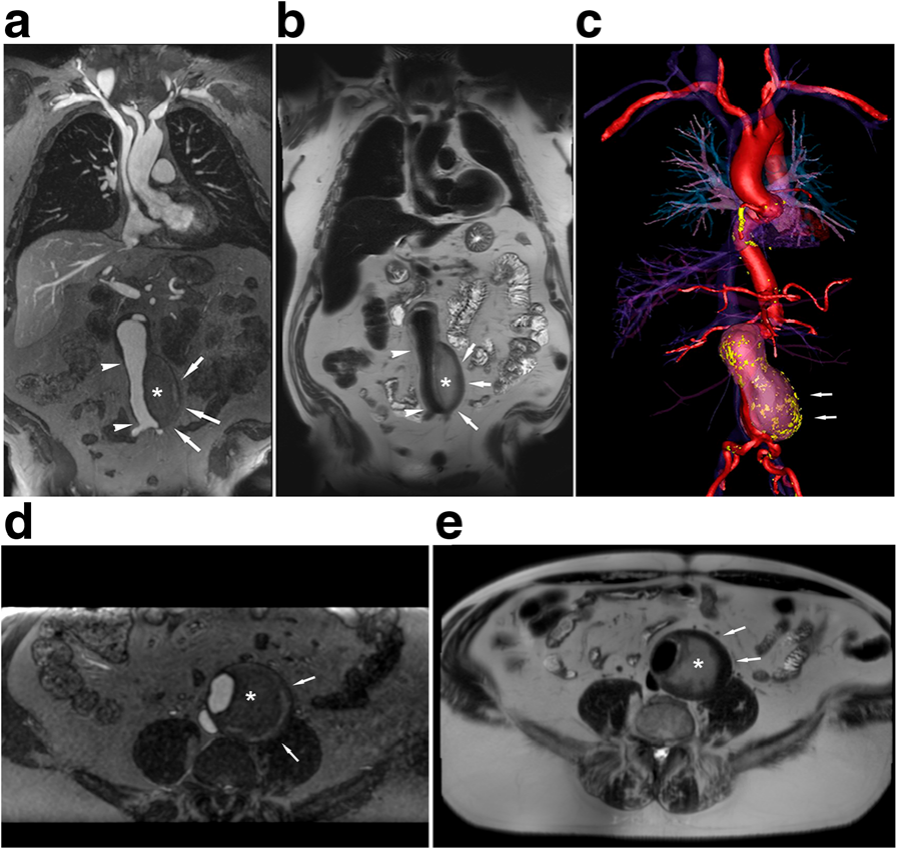
Ferumoxytol enhanced (FE) coronal and axial CMR images (3 T) belonging to a 71-year-old male with an infrarenal abdominal aortic aneurysm post endovascular aorto-bifemoral stent repair. Ferumoxytol CMR was performed to evaluate for endoleak in the setting of renal impairment. Coronal and axial bright blood high resolution CMR angiogram (**a**, **d**) and FE-HASTE (**b**, **e**) images demonstrate a widely patent stent graft (white arrowhead) without endoleak. Note the contrasting intraluminal bright blood signal compared to uniformly dark blood signal suppression in all vascular lumens and intra-cardiac chambers (**b**, **e**, Additional file 2: Video S2). Thin aortic valve leaflets are clearly depicted (**b**). FE-HASTE depicts thrombosis of the aneurysm sac (asterisk, **b** and **e**), which is located outside of the endograft. 3D color volume rendered bright blood CMR angiogram (**c**, Additional file 3: Video S3) illustrates the relationship between the thrombosed aneurysm sac (white arrows) and the endograft

**Fig. 6:**
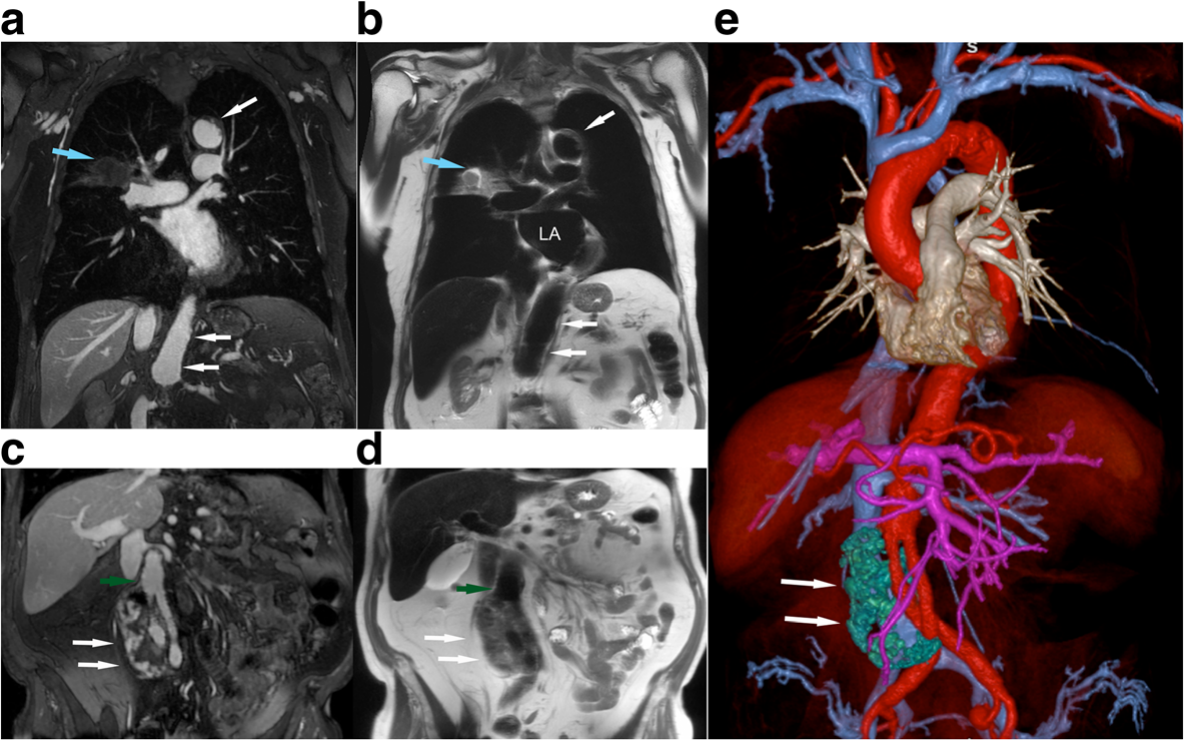
Post-ferumoxytol 3D CMR angiogram multiplanar reformat (**a**, **c**) and T2 FE-HASTE (**b**, **d**) coronal CMR images (3 T) belonging to an 85-year old male with infrarenal abdominal aortic aneurysm post endovascular aorto and bilateral iliac stent graft repair. The CMR exam was performed to evaluate for endoleak. There is diffuse atherosclerosis of the transverse and abdominal aorta with eccentric and ulcerated plaques (**a**-**b**, white arrow; Additional file 4: Video S4). An incidental heterogeneous lesion with serpiginous border (**a**-**b**, blue arrow) is present in the right middle lobe and is well demarcated on FE-HASTE. Coronal FE-HASTE (**b**, **d**) demonstrates uniformly suppressed blood signal throughout the lumen. The aneurysm sac (**c**-**e**, white arrows) has circumferential contrast leakage (**c**-**d**), suggestive of a Type II endoleak. Relationships between the endoleak (**e**, white arrow, segmented green) and stent grafts are depicted on the 3D color volume rendered bright blood CMR angiogram image (**e**). Compared to the bright blood CMR angiogram image (**c**, green arrow), the intra-luminal blood signal is completely suppressed in the patent iliac stent graft (**d**, green arrow). Comparison of aortic vessel wall characteristics and endoleak tissue characteristics on FE-HASTE and bright blood 3D CMR angiogram is available as Additional file 5: Video S5. LA, left atrium; MPR, multiplanar reformat

Two patients with abdominal aneurysms post endovascular repair are presented in Figs. 5 and 6. In both examples, the complementarity of black blood FE-HASTE and bright-blood 3D CMR angiography was helpful for demonstrating stent patency, characterizing the aneurysm sac, and determining whether an endoleak was present. In Fig. 5, FE-HASTE allowed for high contrast discrimination between perfused lumen and thrombus. The lack of enhancement within the aneurysm sac suggests the absence of an endoleak. Conversely, the combination of FE-HASTE and bright-blood CMR angiogram in Fig. 6 showed near circumferential contrast leakage into the aneurysm sac, suggesting a Type II endoleak. The complementarity of FE-HASTE and bright-blood CMR angiogram also allowed for confident identification of diffuse, eccentric, and ulcerated atherosclerotic plaques along the aorta. Notable is the uniform and complete blood signal suppression depicted on the FE-HASTE images.

**Fig. 7:**
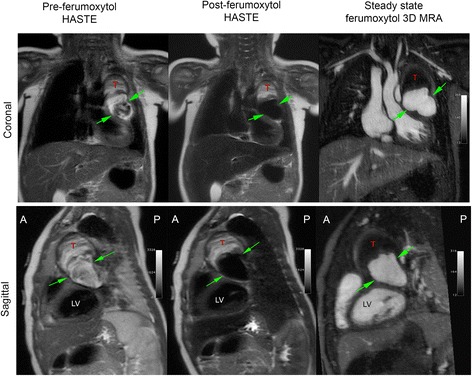
Pre- and post-ferumoxytol CMR images (3 T) belonging to a 4-year-old girl with Kawasaki disease. CMR was performed to evaluate left and right coronary aneurysms. Compared to pre-ferumoxytol HASTE images, excellent blood signal suppression of intracardiac chambers and vascular segments is demonstrated on FE-HASTE images. The thrombosed portion of the left coronary aneurysm (T) and the lumen (green arrows) are well-demarcated on FE-HASTE and 3D high resolution CMR angiogram images. The distinction between the partially thrombosed coronary aneurysm and the perfused lumen is not well depicted due to complex flow patterns on pre-ferumoxytol HASTE

**Fig. 8:**
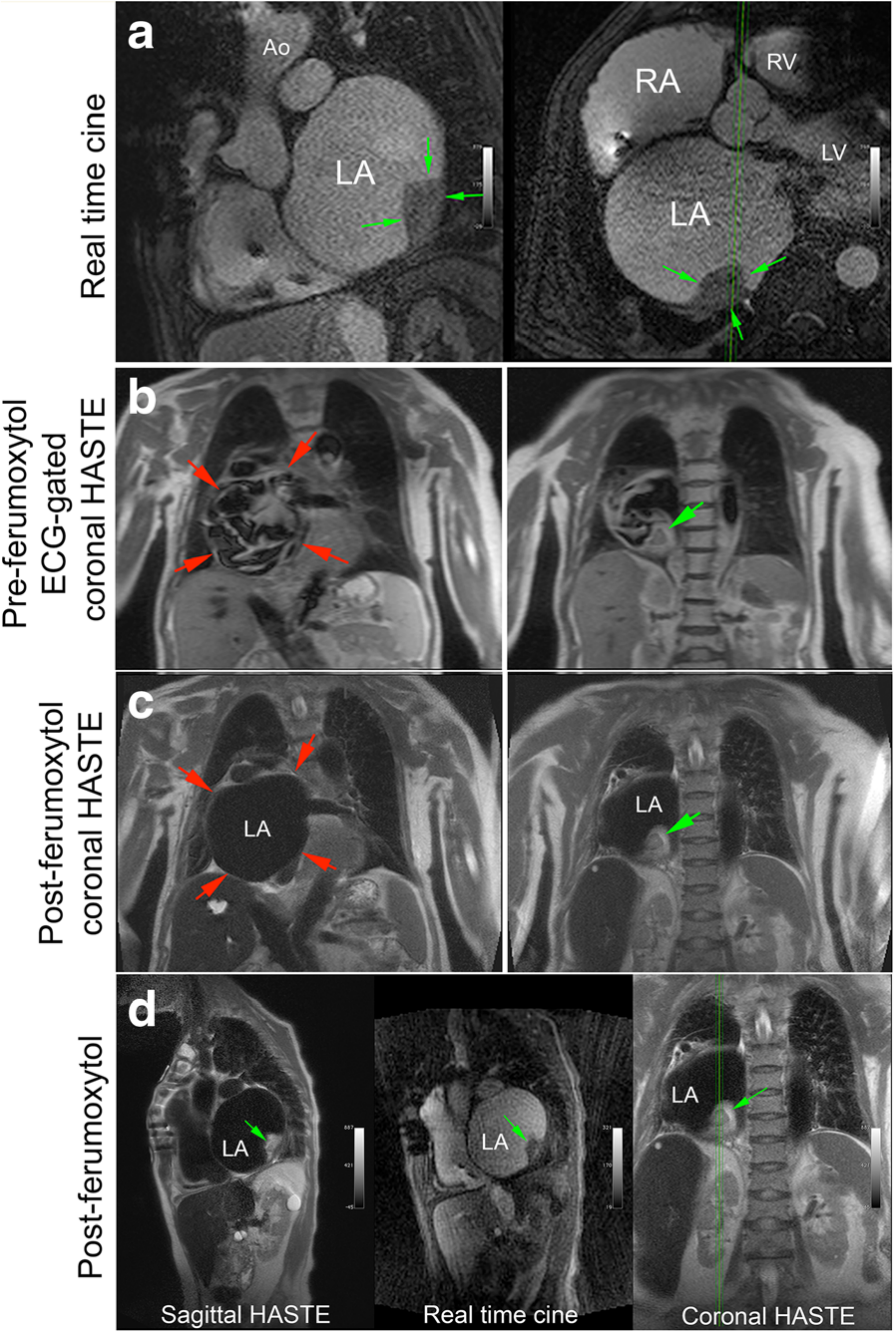
Pre-ferumoxytol ECG-gated HASTE and post-ferumoxytol HASTE CMR images without ECG-gating (3 T) belong to a 71-year-old female with dilated cardiomyopathy and bouts of sustained ventricular tachycardia (VT). FE-CMR was ordered to delineate a possible left atrial (LA) mass prior to undergoing VT ablation. Real-time cine FE-CMR images (panel **a**) demonstrate a large, sessile, immobile mass (green arrows) in the posterolateral wall of a severely dilated left atrium (red arrows). Compared to ECG-gated pre-ferumoxytol HASTE imaging (panel **b**), FE-HASTE CMR (panel **c** and **d**) achieved more complete and homogenous blood signal suppression, which enabled confident and definitive diagnosis of a left atrial mass (green arrows). The morphologic appearance of the left atrial mass on FE-HASTE CMR correlated well with findings on bright-blood real-time cine imaging (panel a; panel d, center image). Ao, aorta; LV, left ventricle; RV, right ventricle

The effectiveness of FE-HASTE is further illustrated in Fig. 7 and Fig. 8. In Fig. 7, FE-CMR was performed to evaluate known coronary aneurysms in a 4-year old child with Kawasaki disease. On pre-ferumoxytol HASTE images, the thrombosed portion (Fig. 7, red T) of the left coronary artery aneurysm and the perfused lumen have similar signal. However, blood signal suppression on FE- HASTE clearly differentiated the perfused lumen from the superior thrombosed portion (Fig. 7, green arrows). These findings correlate closely with the bright-blood CMR angiogram. In the setting of cardiomegaly and very slow flow, FE-HASTE images provided more reliable and complete blood signal suppression in the left atrium (Fig. 8). ECG-gating during pre-ferumoxytol HASTE imaging (Fig. 8, panel b) failed to provide effective blood signal suppression whereas FE-HASTE succeeded fully, even without ECG-gating.

### Quantitative analysis

Compared to pre-ferumoxytol HASTE images, intra-luminal blood signal suppression was more effective on FE-HASTE (Table 3). The median homogeneity index for FE-HASTE images was at least five times lower than pre-ferumoxytol HASTE images (*p* < 0.001), indicating more effective signal suppression post-ferumoxytol. Similarly, the mean SNR for all ten cardiovascular regions (superior vena cava, both atria, both ventricles, pulmonary trunk, right and left pulmonary arteries, ascending aorta, and descending thoracic aorta) was significantly lower for FE-HASTE images (5.6 [IQR 3.2–10.0] vs 21.5 [IQR 12.5–39.4], *p* < 0.001). Comparison of SNR for each region is outlined in Table 3. The CNR for FE-HASTE images (28.3 [IQR 16.6–46.8]) was higher than pre-ferumoxytol (20.5 [IQR 10.9–42.9]), but this was not statistically significant (*p* = 0.14).

**Table 3:** Comparison of SNR measurements as a proxy for uniform intra-luminal blood signal suppression (*n* = 93)

	Pre-FE HASTE DIR	Post-FE HASTE	*P* value
Image noise	10.0 (6.7–16.5)	9.7 (6.2–15.1)	0.344*
Homogeneity index	108.0 (65.0–170.4)	24.5 (18.0–32.8)	<0.001*
Signal-to-noise ratio (SNR)			
Overall	21.5 (12.5–39.4)	5.6 (3.2–10.0)	<0.001**
SVC	11.2 (5.6–23.0)	6.2 (3.5–13.0)	<0.001**
RA	11.3 (5.1–23.6)	3.3 (2.2–6.2)	<0.001**
RV	6.4 (2.7–12.3)	2.7 (1.6–6.0)	<0.001**
LA	27.5 (10.8–47.0)	4.3 (2.4–7.1)	<0.001**
LV	22.4 (5.1–44.3)	3.7 (2.0–5.6)	<0.001**
MPA	18.5 (8.1–38.0)	6.2 (2.9–10.9)	<0.001**
RPA	22.3 (6.9–37.2)	5.4 (2.9–12.3)	<0.001**
LPA	22.0 (8.0–47.1)	6.1 (3.4–11.7)	<0.001**
Asc Ao	12.4 (5.0–29.6)	5.1 (2.9–9.2)	<0.001**
Dsc Ao	33.2 (15.3–67.6)	6.9 (3.4–15.1)	<0.001**

Values are reported as median and interquartile range

*Asc Ao* ascending aorta, *DIR* double-inversion recovery, *Dsc Ao* descending aorta, *FE* ferumoxytol enhanced, *LA*, left atrium, *LPA* left pulmonary artery, *LV* left ventricle, *MPA* main pulmonary artery, *RA* right atrium, *RPA* right pulmonary artery, *RV* right ventricle, *SNR* signal-to-noise ratio, *SVC* superior vena cava

*Wilcoxon rank sum test; **ANOVA for repeated measures

## Discussion

Our study demonstrates that FE-HASTE is a simple and effective technique that overcomes many limitations associated with conventional black-blood imaging. FE-HASTE produced reliable, flow independent, uniform suppression of blood signal in the vascular lumen and cardiac chambers. Compared to pre-ferumoxytol HASTE, FE-HASTE yielded more consistent and greater homogeneity in blood signal suppression. In conventional black-blood imaging, the effectiveness of dual inversion is attributable to the longer duration available for inflow of the out-of-slice inverted spins into the imaged volume. With the use of ECG gating and appropriate adjustment of the inflow time within the cardiac cycle, the effectiveness of blood signal suppression can be increased. However, the requirement for cardiac gating imposes limits on the duration of the image acquisition window and on spatial resolution, particularly for slow flow where the inflow time may occupy a substantial portion of the cardiac cycle. Further, ECG gating at 3 T can be problematic and prone to inconsistency.

In our study, the dual inversion module of the pre-ferumoxytol HASTE images was in most cases positioned randomly within the cardiac cycle and this may help explain the frequent persistence of intravascular signal. It is well recognized that with pulsatile blood flow, the image acquisition window may encompass anywhere from maximum velocity to zero velocity flow on various slices. Therefore, ours was not a comparison between an optimal pre-ferumoxytol dual inversion HASTE and FE-HASTE, but rather a study of how well FE-HASTE, with parameters chosen to provide high resolution images and prevent inter-slice cross-talk, supports complete blood signal suppression without any magnetization preparation schemes. That said, even with ECG gating, dual inversion black blood imaging may fail if timing is not optimal or if flow is very slow, as we found in the patient illustrated in Fig. 8.

Blood signal suppression with ferumoxytol is independent of blood flow because, beyond a threshold TE, the blood signal will decay because of its T2. In our study, the TEs employed at both 1.5 T and 3 T were clearly at or beyond the minimum to ensure full blood signal suppression, although we did not explore what the threshold minimum TE was. Our study confirmed that, with appropriate T2 weighting, FE-HASTE can be leveraged to complement bright-blood CMR for characterization of cardiovascular pathology.

Multiple strategies have been explored for blood signal suppression in MRI, including spatial presaturation, long train radiofrequency pulses such as RARE (rapid acquisition with relaxation enhancement) [10], inversion recovery magnetization preparation techniques [4, 11], or T2* shortening of deoxygenated blood (susceptibility weighted imaging). Other black-blood techniques and their variants such as presaturation of inflowing blood by slab-selective radiofrequency pulses [12], motion-sensitizing magnetization preparation gradient echo sequences [13], multislice motion-sensitized driven-equilibrium (MSDE) turbo spin-echo sequences [14], free breath 3D whole heart MSDE [15], and radial steady-state free precession [16] have been also been introduced. Despite these efforts, black-blood imaging remains challenging in clinical practice due to troublesome artifacts and flow dependency. Frequently, sufficient image quality can be achieved through selection of imaging parameters on an individualized patient basis and is influenced by the skill and experience of clinicians and technologists. With FE-HASTE, customized imaging parameters and magnetization preparation schemes are not required for effective blood signal suppression, and less restrictive parameters can be prescribed to optimize other aspects of image quality. Based on our findings, FE-HASTE is a simple strategy for discrimination between flow artifact and true vessel wall pathology such as thrombus, tumor, or plaque.

Although ferumoxytol is FDA approved for treatment of iron deficiency anemia in adults with kidney disease, it has several promising attributes for diagnostic CMR imaging [5, 6, 17, 18]. First, unlike gadolinium-based contrast agents (GBCAs), ferumoxytol is not cleared by the kidneys, but is metabolized by the reticuloentholelial system and the iron core is incorporated into the body for biologic use. Second, although the longitudinal *r*_1_ relaxivity of ferumoxytol is similar to gadofosveset, its intravascular half-life is substantially longer (> 15 h). With a molecular weight of 750 kD and particle size of 30 nm, ferumoxytol has excellent fidelity as an intravascular contrast agent with both strong T1 and T2* shortening effects [7]. The dual potential of ferumoxytol was previously highlighted by Li et al. [19] in a pilot study of lower extremity deep venous thrombosis. With the exception of an abstract by Vu AT et al. describing ferumoxytol black-blood cine imaging [20], the more general use of ferumoxytol as a black-blood imaging agent has not previously been described.

A recent FDA warning highlighted the occurrence of rare, but serious hypersensitivity reactions associated with the therapeutic use of ferumoxytol [21], some of which were associated with fatal outcomes. Based on therapeutic use data from post-marketing trials [22–26], the calculated aggregate risk of anaphylactic reaction is 0.03%. Suggestions to minimize hypersensitivity reactions include dilution and slow infusion. Monitoring of vital signs up to 30 min post-infusion is also recommended and should be standard practice. Compared to other USPIOs, the outer carbohydrate shell of ferumoxytol was designed to have lower free-iron release [27], decreased immunologic allergic reaction, and an improved safety profile [28]. To date, no severe adverse events have been directly associated with the diagnostic use of ferumoxytol CMR in both small and large cohort single-center safety studies [9, 29–31].

Our study has several limitations. First, although images were evaluated in a blind and random way, the apparent difference in the uniformity of the blood signal suppression on FE-HASTE images made it challenging to truly blind the readers. Second, not all images received perfect image quality scores even though blood signal suppression was excellent on FE-HASTE images. This observation may relate to the intrinsic limitations of HASTE imaging, and also the lack of cardiac gating which may result in occasional blurring along vessel or endocardial borders. However, black-blood imaging is rarely used alone, but in conjunction with bright blood imaging techniques, which is where the complementary duality of FE imaging may be of considerable value. Our study was also not designed to assess the diagnostic accuracy of FE-HASTE, but rather to gauge its effectiveness for flow independent blood signal suppression. More extensive work will be required to address the broader question of diagnostic accuracy. Lastly, because of ferumoxytol’s long intravascular half-life, imagers need to be aware that ferumoxytol may influence CMR tissue contrast for days or weeks, and should interpret follow up studies appropriately. While the issue of iron overload has been raised, this is rarely a practical clinical concern, because iron overload is a long-term process and is uncommon other than in hemochromatosis. Moreover, the majority of the world’s population is iron deficient [32, 33] and the amount of iron available for physiologic use is tightly regulated [34, 35]. Nevertheless, careful screening and appropriate candidate selection are encouraged.

## Conclusions

FE-HASTE imaging provides a simple, fast, and reliable strategy for uniform, flow independent blood signal suppression at 3 T and 1.5 T. Compared to pre-contrast HASTE, FE-HASTE obviates the need for magnetization preparation schemes and ECG gating, which add complexity and may limit achievable resolution. In those already undergoing clinical FE-CMR for bright-blood applications, black-blood FE-HASTE is a simple and practical technique that can be used in conjunction with bright-blood imaging for further characterization of cardiovascular pathology.

## Additional files

Additional file 1: Video S1. Coronal multi-slice comparison of pre-ferumoxytol HASTE and ferumoxytol-enhanced HASTE images. (MOV 3479 kb)

Additional file 2: Video S2. Coronal multi-slice ferumoxytol-enhanced black blood HASTE in a patient with a repaired infrarenal abdominal aortic aneurysm. (MOV 2226 kb)

Additional file 3: Video S3. 3D color volume-rendered bright blood ferumoxytol-enhanced CMRA of a thrombosed aneurysm sac and endograft. (MOV 8820 kb)

Additional file 4: Video S4. Coronal multi-slice ferumoxytol-enhanced black blood HASTE in a patient with infrarenal abdominal aortic aneurysm and bilateral iliac stent graft repair. (MOV 1961 kb)

Additional file 5: Video S5. Comparison of aortic vessel wall characteristics and endoleak tissue characteristics on ferumoxytol-enhanced HASTE and bright blood 3D CMRA. (MOV 4526 kb)

## Abbreviations

Ao: Aorta
CMR: Cardiovascular magnetic resonance
CNR: Contrast-to-noise ratio
DICOM: Digital Imaging and Communication in Medicine
ECG: Electrocardiogram
FDA: United States Food and Drug Administration
FE: Ferumoxytol-enhanced
GBCA: Gadolinium-based contrast agent
HASTE: Half-Fourier Acquisition Single-shot Turbo Spin-echo
LA: Left atrium
LPA: Left pulmonary artery
LV: Left ventricle/left ventricular
MPA: Main pulmonary artery
RA: Right atrium
RPA: Right pulmonary artery
RV: Right ventricular
ROI: Region-of interest
SI: Signal intensity
SNR: Signal-to-noise ratio
USPIO: Ultrasmall superparamagnetic iron oxide
